# Introducing students to neural communication: an embodied-learning classroom demonstration

**DOI:** 10.1038/s41539-020-00077-1

**Published:** 2020-12-04

**Authors:** B. D. M. Hatin

**Affiliations:** School of Education and Social Sciences, University of the West of Scotland, High Street, Paisley Renfrewshire, PA1 2BE UK

**Keywords:** Education, Psychology, Synaptic transmission

## Abstract

Learning about neural communication can be a dry and challenging undertaking, particularly for students without a background in biology. To enhance learning of this and other STEM material, there has been a call for science educators to embrace the use of active learning techniques. The aim of this Brief Communication is to encourage the use of embodied metaphors in the university classroom by sharing an active learning method for introducing students to a number of key concepts in neural communication. The students work in pairs or small groups, using foam projectiles such as Nerf guns to work through several metaphors for electrical and chemical processes including action potentials, neurotransmission and receptor action, excitatory and inhibitory post-synaptic potentials and neurotransmitter inactivation. The activities are easy to stage and lend themselves well to customisation based on available class size, classroom space, and resources. Student feedback showed that the activities improved self-reported impressions of understanding and ability to convey key concepts to others. The activities thus can serve as a useful method of student engagement and help develop understanding of complex material in a neuroscience classroom.

## Introduction

The curriculum for most introductory psychology courses (and certainly for biopsychology courses) involves introducing undergraduate students to the biological underpinnings of neural communication. Instructors sometimes use the aid of diagrams and videos to help demonstrate concepts such as graded potentials, action potentials, saltatory conduction, exocytosis, and neurotransmitter inactivation. However, the traditional method of teaching these concepts via a lecture may not be the most effective way for students to learn^1^. Active learning refers to a broad method of classroom instruction that involves student engagement, allowing for meaningful thought about the subject as learning activities are completed^2^. This can include any number of independent or group activities that involve higher-order thinking such as reading and writing, discussing and problem solving^3^. In so doing, it allows students to construct their own understanding of the subject, as opposed to passively receiving information from an instructor^1^. In a meta-analysis of studies that compared undergraduate student performance in STEM classes with traditional versus active learning, Freeman et al.^1^ found that active learning activities such as group problem solving, worksheets and use of personal response systems, improved examination scores and lowered failure rates^1^.

One challenge particular to STEM and neuroscience classrooms is that much of the material is abstract in nature. For example, the chemical activity that takes place at the synapse cannot be directly viewed by students, nor can receptor activation. However, research suggests active and creative learning that is rich with metaphors can shape understanding of abstract science concepts^4,5^. Thus, the use of the “lock and key” metaphor that is often used in lessons on receptor activity is one example of a common method to enhance student understanding. While metaphors can be useful, whole-body engagement in a learning environment supports and improves learning beyond what is observed through passive engagement with a metaphor^6^. For example, acting out the opening of a lock with a key would be more effective than passively hearing the metaphor. This is what is known as an embodied metaphor, and may be a type of active learning especially well-suited to abstract neuroscience topics.

Although STEM educators are calling for the development of more active learning techniques^7^, there are currently minimal resources available to illustrate the concepts of neural communication in an active, let alone embodied, manner. There are a few notable examples, most of which are aimed at a public audience or a primary or secondary level, rather than university students^8–11^. For example the iNeuron app is aimed at secondary-level students; it is a game-based learning activity wherein students complete challenges to create neural circuits^12^. The first two levels are free and educators may pay a modest fee to unlock the full game for their classrooms.

The use of embodied learning is currently even more limited in neuroscience. One key aspect of neural communication, the action potential, has been explored via the use of an embodied metaphor in a university classroom^13^, which was reported to improve student understanding of the stages of an action potential. However, there are a number of other key processes besides the action potential that can be explored through embodied learning. The following activities will allow instructors to engage their students in an active and embodied manner, using simple and inexpensive methods to exemplify different aspects of neural communication. In doing so, students can grasp a deeper understanding of the material.

## Results

### Preliminary student feedback

The activities, described below in the Methods section, were carried out in a neuroscience classroom at the University of the West of Scotland in September 2019. After a period of 8 weeks, students were asked to provide feedback on the memorability, usefulness and efficacy of the activity. Ethics approval was granted by the University of the West of Scotland’s School of Social Science and Education Ethics Committee. Students were provided with a link to an online survey, wherein they first provided digital informed consent in lieu of written consent. Then they answered questions regarding their impressions of the activity. A total of 17 students provided feedback, rating statements from 1 (completely agree) to 5 (completely disagree). According to the averaged student ratings, they highly agreed that the activities were memorable (*M* = 1.23, SD = 0.43), that it helped them learn more than they would have by just listening to the lecture (*M* = 1.88, SD = 1.22), and that they would recommend using the activity for future groups of students (*M* = 1.59, SD = 1.71).

### Additional student feedback

**Fig. 1:**
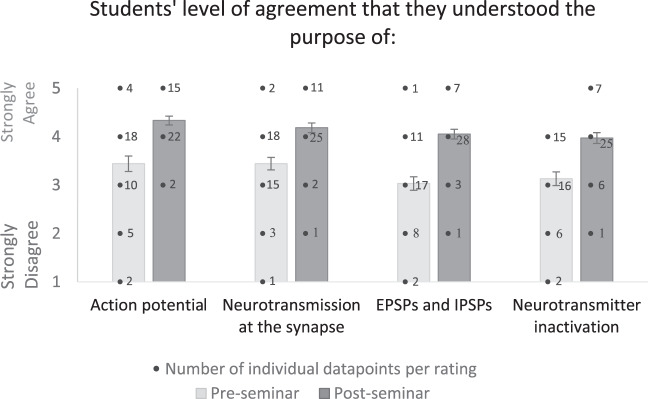
Pre- and post-seminar student ratings (with s.e.m. error bars) on how well they understood the purpose of key concepts of neural transmission. The bar graph indicates average ratings, and the overlayed numbers provide counts of individual data points.

**Fig. 2:**
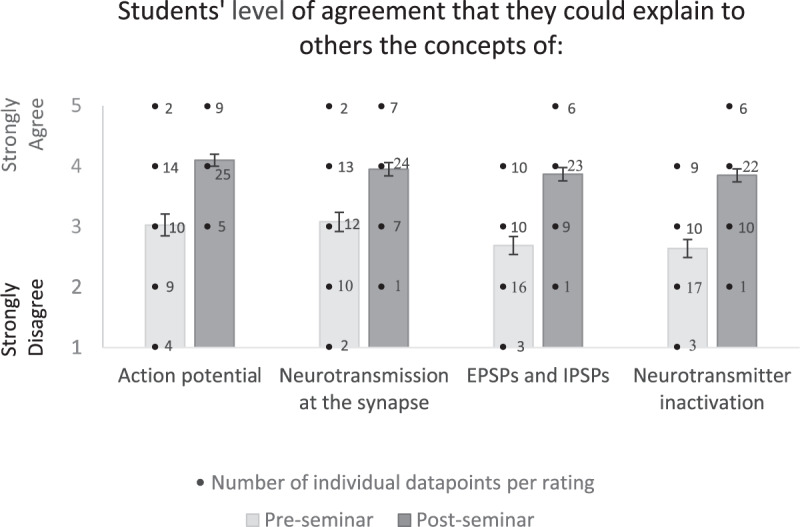
Pre- and post-seminar student ratings (with s.e.m. error bars) on how well they felt they could explain key concepts of neural transmission to others. The bar graph indicates average ratings, and the overlayed numbers provide counts of individual data points.

The activities were carried out a second time in February 2020 and levels of understanding were measured in a pre-post design. Students attended a traditional 2-h lecture on neural communication and then voluntarily completed an online survey on their understanding of the four key concepts. Students were provided with a link to the survey during a 1-h post-lecture break, wherein they first provided digital informed consent in lieu of written consent. Following this they participated in the activities in a 1-h seminar session, and then voluntarily completed the survey once again post-seminar. A total of 39 students completed both surveys, and all responses were made on a scale of 1 (strongly disagree) to 5 (strongly agree). As shown in Figs. 1 and 2, the active learning activities increased how well students felt they understood the purpose of these key concepts, and also increased how well they felt they could explain these key concepts to others. This may suggest improved recall and confidence with the topic, and at the least demonstrates improved self-efficacy^13^. Analyses were run to examine whether these differences were statistically significant. Visual examination of boxplots revealed that there were a total of 14 outliers, but further inspection indicated that these were primarily students whose level of understanding of certain concepts was very poor or very high. No outliers were removed from analyses. Results of two-tailed paired-sample *t*-tests demonstrated that all pre-post comparisons were statistically significant, *t*(38) = 5.49–7.60; all *p*s < 0.001. Anonymized rating data are available in Supplementary Tables 1 and 2.

Open-ended questions from both activity sessions showed that students reported that strengths of the activity were that “it helped to make a complicated process simpler” and that it was “fun”, “interesting”, “interactive” and “new”. Weaknesses revolved around the shyness or reluctance of some classmates to fully participate. While this is a common issue with group activities, it can be mitigated by ensuring each student has a clearly defined role, and from an anecdotal perspective by also ensuring each student interacts with the Nerf toys before the start of the activities (e.g., by having a quick icebreaker activity wherein each student in each group fires the Nerf gun once; the first group to finish wins). One student suggested ending the session by writing short paragraphs about each activity and how the metaphors represent the neural activity.

## Discussion

The activities were designed to help instructors introduce students to four different aspects of neural communication in an active learning environment. Students and their foam projectiles take on a variety of roles depending on the context, including as a soma, axon hillock, presynaptic axon terminal, post-synaptic cell, and metabotropic and ionotropic receptors. In doing so, the students are using embodied metaphors, which have been demonstrated to enhance learning^5^. Active learning techniques such as the ones described in the Methods section are especially important in a science classroom, wherein many concepts are abstract and complex^13^. Research has shown that active learning is highly effective for subject matter in the STEM fields^7^, improving student grades and increasing their feeling of understanding^1^. The present Brief Communication provides instructors with a way to use active learning in their own classrooms when introducing students to neural communication.

Although the activities may work better in a relatively small classroom setting, they can be modified as appropriate based on class size, resources and space. For example, if there are minimal foam projectile toys, or a large number of students, then a different group of student volunteers could be asked to model each activity for the rest of the class, one at a time. However, for a larger class where not all students can participate, this will not encourage active learning in those who are passively observing. Alternately, activity stations could be set up around the classroom with written instructions so that students can try out each activity at their own pace. The activities can also be modified to encourage deeper critical thinking, wherein students could be guided through the activity and then asked to figure out how their given task fits with each key concept (rather than having the instructor point out the metaphors). Alternately, students may even come up with their own metaphors/activities. Regardless of the format, the activities can be easily completed within 30 min.

A final consideration that educators may wish to take into account when deciding whether and how to employ these learning activities is the current socio-political climate and historical context with respect to the use of weapons. While the foam projectiles are a common children’s toy that do not cause physical harm, the idea of using toy weapons may not be appropriate in some instances, such as locations where gun-related violence is more common. In this respect, a discretionary warning may be given to students prior to the activity, or alternative activities may be considered (e.g., by modifying materials developed to teach neuroscience concepts to children or the public, such as the BrainU website mentioned below).

In the future, educators may wish to take the activities a step further and consider making links between the act of learning and its underlying neurobiological activity. Indeed, by participating in these embodied-learning activities, students are effectively reshaping their own synapses and strengthening neural pathways. Concepts of neuroplasticity and learning could easily be linked to, and fed forward from, the activities. Along a similar line, Dubinsky et al.^14^ wrote about developing research-informed teachers through their BrainU professional development institute, wherein teachers come to recognise that they are “designers of experiences that ultimately change students’ brains” (p. 318). Their BrainU website has some resources that educators can use to teach neuroscience concepts in a research-informed manner, some of which may be modified to be used in a university neuroscience classroom.

The present paper was focused on sharing an easy and low-budget embodied-learning activity that students reported enjoying and found to facilitate deeper learning. The activities are geared towards an introductory university lesson, and can lay the groundwork for more complex discussion of cellular processes. Informal evaluation of the activity’s efficacy has shown that students ask more questions and express that the activity is memorable and helpful. In addition, a pre-post survey demonstrated improvements in self-reported understanding of neural communication following completion of the activities. Future research may also wish to explore the efficacy of these particular embodied learning activities using tests of knowledge rather than self-reported impressions of understanding. The author hopes that neuroscience educators find the suggested activities to be a useful starting point to introduce more active and embodied learning into their own classrooms and enhance student understanding of neural communication.

## Methods

### Study design

Ethics approval was granted by the University of the West of Scotland’s School of Social Science and Education Ethics Committee. Students were provided with a link to an online survey, wherein they first provided digital informed consent in lieu of written consent.

Prior to the active learning session, the instructor should provide a basic overview of the chemical and electric aspects of neural communication. Following this, the instructor can work through the following four activities with students to further explore and reinforce the material. In advance of the lesson, the instructor can ask students to bring along foam projectiles such as Nerf guns, and the instructor should supply a number of Nerf guns as well (some of which fellow colleagues may be able to contribute).

For the active learning session, students should be divided into groups of a minimum of three people each. Larger groups can be formed depending on the class size and number of available foam projectiles. The instructor will introduce and guide students through each activity, then follow up with a discussion of how the activity links back to each key concept.

### Key concept 1: action potentials

Simon-Dack^13^ provided an excellent active learning technique to introduce students to the processes of an action potential, to which the reader is referred^13^. In the context of the present activity, students can be more broadly introduced to the concept of a firing threshold, the all-or-none principle and the refractory period. First, students should be asked to fire the Nerf gun as strongly as possible, then as weakly as possible. The students should come to realise that no matter how hard or gently they pull the trigger, once they hit the threshold the toy will shoot its projectile at the same speed. The instructor should then relate this to the firing threshold of a neuron, noting that the moment the threshold was reached (the trigger reached just past the point of resistance), the cell produced an action potential (the foam projectile was fired). The Nerf gun (the neuron) could either fire or not fire, akin to the all-or-none principle. The trigger also can be likened to the axon hillock. Additionally, students will need to reload the gun before they can fire again, and even with a rapid-fire toy, the chamber will need a moment to rotate and load the next projectile before it can fire. This somewhat represents the refractory period: the cell could “fire” again, but it takes more effort to do so and there is a maximum rate of fire that cannot be exceeded.

### Key concept 2: neurotransmission

With this activity, students will emulate the release of neurotransmitters across the synaptic cleft, along with two types of receptor action. For the first part, one member of each group (Student 1) should fire the foam projectile towards another group member (Student 2). This group member, once hit, can emulate opening a door. The instructor can then describe how the student holding the Nerf gun (Student 1) represented the axon terminal of the presynaptic neuron, and the Nerf gun is acting as a vesicle full of neurotransmitters. When they fired the toy gun they triggered the process of exocytosis, wherein the vesicle releases its contents into the synaptic cleft (the gap between the first and second student). Student 2 represented the post-synaptic cell (and the receptors therein). Furthermore, when Student 2 was hit and emulated opening a door, this was akin to a neurotransmitter (foam projectile) activating an ionotropic receptor (Student 2), which directly opened a channel allowing ions to enter the cell (when the student emulated opening a door).

For the second part of this activity, the same task should be repeated (and students may switch roles), but this time Student 2 should link arms with another group member, Student 3. When Student 2 is hit, they should ask Student 3 to open the door. Student 3 can then unlink arms and walk to a “door” to open it. The instructor can then discuss how this demonstrated the different actions undertaken by metabotropic receptors: when a neurotransmitter molecule activated a metabotropic receptor (Students 2 and 3 linked together), a channel still opened. However, this happened more indirectly, such that a G-protein (Student 3) broke away from the metabotropic receptor and opened up the channel.

### Key concept 3: excitatory and inhib**i**tory post-synaptic potentials

Following from key concept 3, students should be directed to the “door” (channel) which they have opened. The instructor can note how ions can flow into and out of this channel, and depending on the type of ion, this can make the cell more or less likely to fire. Students should then work with their group members to find ways to make the Nerf gun more or less likely to fire. For example, they might note that moving a finger to the trigger represents excitatory post-synaptic potentials (EPSPs), whereas pulling the finger away represents inhibitory post-synaptic potentials (IPSPs). Similarly, group members shouting to pull the trigger represents EPSPs whereas peer pressure to put the toy down can represent IPSPs.

### Key concept 4: neurotransmitter inactivation

Finally, the gate must close and receptor action must stop, so this final activity will explore neurotransmitter inactivation. First, students can be guided to fire a foam projectile, and then pick up and reload that same projectile as quickly as possible. This represents reuptake of the neurotransmitter back into the presynaptic cell. Next, students can fire a foam projectile and another team member will tear or cut up the projectile, or emulate doing so, to represent degradation. Third, students can fire a projectile and then a team member can roll it out of the way of their group, representing the diffusion of the neurotransmitter away from the synaptic cleft. Finally, students can fire the projectile and another team member can pick it up and throw it in a litter bin, representing the role of glial cells in cleaning the synaptic cleft and breaking down the neurotransmitter.

### Reporting summary

Further information on research design is available in the Nature Research Reporting Summary linked to this article.

## Supplementary information

Supplementary Tables

Reporting Summary

## Data Availability

All data are available upon reasonable request.

## References

[1] Freeman S (2014). Active learning increases student performance in science, engineering, and mathematics. Proc. Natl Acad. Sci. USA.

[2] Prince M (2004). Does active learning work? A review of the research. J. Eng. Educ..

[3] Bonwell, C. C., & Eison J. A. A*ctive Learning: Creating Excitement In The Classroom*. ASHEERIC Higher Education Report No. 1 (George Washington University, Washington, DC, 1991).

[4] Anderson RC (2018). Creative engagement: embodied metaphor, the affective brain, and meaningful learning. Mind Brain Educ..

[5] Niebert K, Marsch S, Treagust DF (2012). Understanding needs embodiment: A theory‐guided reanalysis of the role of metaphors and analogies in understanding science. Sci. Educ..

[6] Gallagher S, Lindgren R (2015). Enactive metaphors: learning through full-body engagement. Educ. Psychol. Rev..

[7] Waldrop MM (2015). Why we are teaching science wrong, and how to make it right. Nature.

[8] Chudler EH, Bergsman KC (2014). Explain the brain: websites to help scientists teach neuroscience to the general public. CBE Life Sci. Educ..

[9] Chudler EH, Karikari TK (2015). Neuroscience for kids. J. Undergrad. Neurosci. Educ..

[10] Dubinsky JM (2010). Neuroscience education for prekindergarten–12 teachers. J. Neurosci..

[11] Schotland M, Littman K (2012). Using a computer game to teach young children about their brains. Games Health J..

[12] Schleisman KB (2018). Learning neuroscience with technology: a scaffolded, active learning approach. J. Sci. Educ. Technol..

[13] Simon-Dack SL (2014). Introducing the action potential to psychology students. Teach. Psychol..

[14] Dubinsky JM, Roehrig G, Varma S (2013). Infusing neuroscience into teacher professional development. Educ. Res..

