# Supplementary material for: Introducing students to neural communication: an embodied-learning classroom demonstration
Source: NPJ Sci Learn. 2020 Dec 4;5:18. doi: 10.1038/s41539-020-00077-1 (PMC7718869; doi:10.1038/s41539-020-00077-1)
Supplement: Supplementary file 1 — Supplementary Tables [file 41539_2020_77_MOESM1_ESM.pdf]

Supplementary Table 1. Pre- and post-seminar level of understanding of key concepts*.

Participant	Pre AP	Pre NT	Pre EPSP & IPSP	Pre NT-I	Post AP	Post NT	Post EPSP & IPSP	Post NT-I
1	5	4	4	4	5	4	4	4
2	4	4	3	3	4	4	4	4
3	5	4	4	4	5	4	4	4
4	4	3	3	4	3	2	2	2
5	4	4	4	4	4	5	4	4
6	5	4	5	3	5	5	5	5
7	4	4	3	4	5	5	5	5
8	4	4	4	4	4	4	4	4
9	3	3	2	2	5	4	4	4
10	3	3	3	3	3	3	3	3
11	4	4	3	3	4	4	4	4
12	2	2	2	1	4	4	4	3
13	2	2	2	2	4	4	4	4
14	2	2	2	2	4	4	4	4
15	4	3	4	3	4	4	4	4
16	4	3	2	4	4	4	4	3
17	4	4	4	4	4	4	4	4
18	4	4	4	3	5	5	4	4
19	4	4	3	4	4	4	5	4
20	3	3	3	3	4	4	4	4
21	2	4	1	4	4	4	4	4
22	4	3	4	2	5	5	4	4
23	4	4	3	4	5	4	4	3
24	3	3	3	3	4	4	4	4
25	3	4	3	4	5	5	5	5
26	4	4	3	3	5	5	5	5
27	3	3	3	3	4	4	4	3
28	4	4	4	4	4	4	4	4
29	4	3	4	3	5	4	4	4
30	3	5	2	4	4	5	4	5
31	3	3	3	3	4	4	4	4
32	4	4	4	4	5	5	5	5
33	1	1	1	1	4	4	3	4
34	5	5	3	3	5	5	4	5
35	2	3	2	3	4	4	3	3
36	3	3	3	3	4	4	4	4
37	3	3	2	3	5	3	4	4
38	4	3	3	2	4	4	4	4
39	1	4	3	2	5	5	5	4

*Abbreviations:
AP = action potential
NT = neurotransmission
EPSP = excitatory post synaptic potential
IPSP = inhibitory post synaptic potential
NT-I = neutrotransmitter inactivation

Supplementary Table 2. Pre- and post-seminar level of ability to explain key concepts* to others.

Participant	Pre AP	Pre NT	Pre EPSP & IPSP	Pre NT-I	Post AP	Post NT	Post EPSP & IPSP	Post NT-I
1	5	4	4	4	5	4	4	4
2	4	2	2	2	4	4	4	4
3	4	4	4	4	5	4	3	3
4	4	3	2	3	3	3	3	3
5	4	4	4	4	4	4	4	4
6	4	5	4	2	5	5	5	5
7	2	2	2	2	4	4	4	4
8	4	4	4	4	4	4	4	4
9	1	2	1	1	5	4	4	4
10	3	3	3	3	3	3	3	3
11	4	4	2	2	4	4	3	3
12	2	2	2	2	4	3	3	3
13	2	2	2	2	4	4	4	4
14	1	1	1	1	4	4	4	4
15	4	3	4	2	4	4	4	4
16	2	2	2	2	4	3	2	2
17	3	3	3	3	3	3	3	3
18	4	4	2	2	4	4	4	4
19	4	3	4	4	4	3	4	4
20	3	3	3	3	4	4	4	4
21	3	3	2	4	4	4	4	4
22	4	3	2	2	5	5	4	3
23	2	2	2	2	3	3	3	3
24	2	2	2	2	4	4	4	4
25	3	4	4	4	5	5	5	5
26	4	4	3	3	5	5	5	5
27	3	3	3	3	4	4	4	3
28	4	4	4	4	4	4	4	4
29	3	3	3	3	4	4	4	4
30	3	4	2	2	4	4	5	5
31	3	3	3	3	4	4	4	4
32	4	4	4	4	5	5	5	5
33	1	1	1	1	4	4	3	4
34	5	5	3	3	5	5	4	5
35	2	4	2	2	4	4	4	4
36	2	2	2	2	4	4	4	4
37	2	2	2	2	4	2	3	4
38	3	3	3	3	3	4	4	3
39	1	4	3	2	4	5	5	4

*Abbreviations:
AP = action potential
NT = neurotransmission
EPSP = excitatory post synaptic potential
IPSP = inhibitory post synaptic potential
NT-I = neutrotransmitter inactivation
